# Color stability of CAD/CAM Zirconia ceramics following exposure to acidic and staining drinks

**DOI:** 10.4317/jced.54404

**Published:** 2017-11-01

**Authors:** Marco Colombo, Marco Cavallo, Matteo Miegge, Alberto Dagna, Riccardo Beltrami, Marco Chiesa, Claudio Poggio

**Affiliations:** 1 Department of Clinical-Surgical, Diagnostic and Pediatric Sciences – Section of Dentistry, University of Pavia, Pavia, Italy

## Abstract

**Background:**

The aim of this *in vitro* study was to evaluate the color stability of CAD/CAM Zirconia ceramics following exposure to acidic drink (Coca Cola) and after exposure to staining solution (coffee).

**Material and Methods:**

All the samples were immersed in different staining solutions over a 28-day test period. A colorimetric evaluation according to the CIE L*a*b* system was performed by a blind trained operator at 7, 14, 21, 28 days of the staining process. Shapiro Wilk test and Kruskal-Wallis ANOVA were applied to assess significant differences among restorative materials. Paired t-test was applied to test which CIE L*a*b* parameters significantly changed after immersion in staining solutions.

**Results:**

One week immersion in acidic drink did not cause a perceivable discoloration for all restorative materials (ΔE < 3.3). Subsequent immersion in coffee affected color stability of all Zirconia samples, even if Kruskal-Wallis ANOVA found significant differences among the various restorative materials.

**Conclusions:**

The ∆Es of CAD/CAM Zirconia ceramics after immersion in coffee varied among the products, but color integrity is not affected by contact with acidic drinks.

** Key words:**CAD/CAM restorative materials, CIE Lab, Zirconia ceramics.

## Introduction

The use of aesthetic materials for dental restorations has increased in the last years, probably due to the development of new fabrication techniques and to the improvement of materials, in order to satisfy patients request. Among the available restoration systems, computer-aided design and computer-aided manufacturing (CADCAM) allows rapid production of tooth-coloured restorations both for resin materials and ceramic materials.

Composite resin block materials have been developed and manufactured for CAD/CAM systems since 2000 (1,2). The CAD/CAM composite resin blocks are made by industries with standardized parameters at high pressure and temperature to reach rights properties related to microstructure level. A CAD/CAM hybrid ceramic block is also introduced which is a polymer-infiltrated feldspar ceramic network enriched with aluminium oxide (3). In the last years the use of zirconia restorations has been increased in restorative dentistry owing to good biocompatibility and excellent mechanical properties of zirconia ceramics(4-9). Among the new materials, zirconia has superior mechanical properties and is the material of choice for restorations especially in high stress bearing area (10). For this reason, CAD/CAM systems have been well consolidated in prosthodontics to build zirconia restorations. Zirconia restorations are made with zirconia core layered or pressed with feldspathic porcelain. However, the success of restorations depends not only on mechanical and physical properties, but also on the esthetic appearance (11,12). Tooth-colored restorative materials should feature excellent color match and high color stability during clinical service (13,14). Visual color difference thresholds can be used as a quality control tool and guideline for selecting esthetic materials (15). Restorative materials in oral cavity are exposed to different changes like humidity, temperature, beverage, food and smoking habits. These factors may be associated with discoloration of the dental materials in the oral environment. The color stability of the composites, is due to exogenous and endogenous reasons (16). The exogenous reasons include the influence of staining solutions such as cola and coffee (17). The endogenous reasons include the system of initiator systems, duration of polymerization, resin matrix composition, conversion of the matrix monomers, particle size and hardness and oxidation of the unreacted carbon double bonds (18). The effects of immersion in cola and coffee, through its high potential for staining, is considered a good test to estimate the materials’ tendency to discolor (19). The CAD/CAM composite resin blocks and CAD/CAM hybrid ceramic block were expected to manifest high resistance to discoloration owing to industrially polymerization process.

The aim of this in vitro study was to evaluate and compare the color stability of different CAD/CAM restorative materials following exposure to acidic drink and exposure to staining solution (coffee).

## Material and Methods

-Specimens’ preparation

Four CAD/CAM restorative materials were tested in this study ( Table 1). For each brand, the A2 Vita shade was selected. A total of forty specimens identical in size (5 mm in diameter, 3 mm thick) of each material were designed with CEREC software 4.2 platform.

**Table 1:**
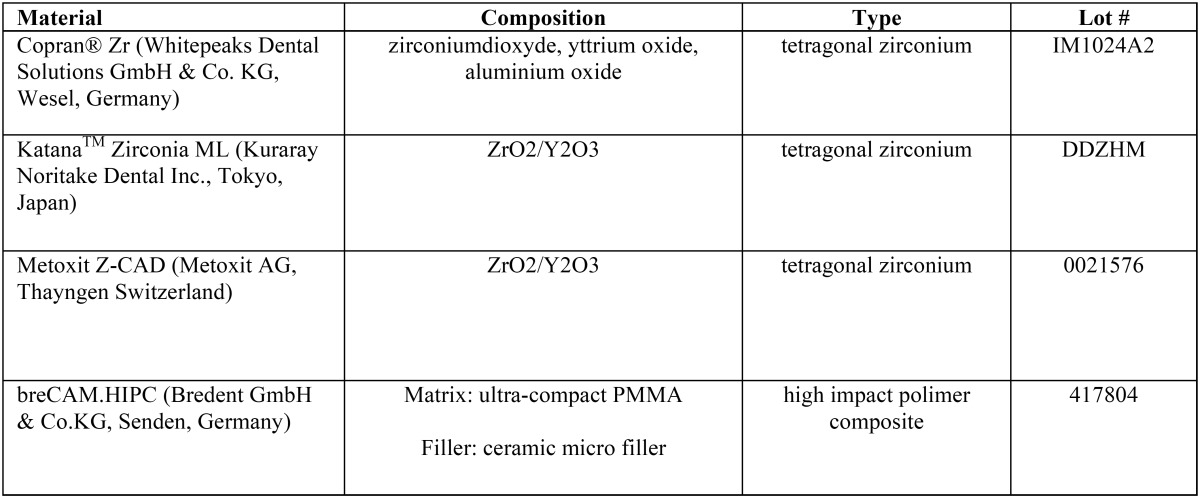
CAD/CAM restorative materials tested in this study.

Specimens of each material were divided into 4 groups (n=10):

• group 1: negative control (physiological solution);

• group 2: immersion in acidic drink (Coca-Cola, The Coca-Cola Company, Milano, Italy) for 1 day + immersion in coffee;

• group 3: immersion in acidic drink (Coca-Cola, The Coca-Cola Company, Milano, Italy) for 1 day + immersion in physiological solution;

• group 4: immersion in physiological solution + immersion in coffee.

-Staining process

The staining solution used was coffee (Nescafe Classic, Nestle, Vevey, Switzerland).

The specimens were immersed in staining solution at room temperature over a 28-day test period. Solutions were changed daily and put in vials with cover that prevent evaporation of staining solutions. Spectrophotometric analysis was made immediately after light-polymerization (T0) and at 7 (T1), 14 (T2), 21 (T3), 28 (T4) days the beginning of the experimentation.

-Color testing

**Figure 1:**
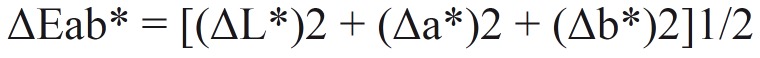
Formula.

For all groups, colorimetric evaluation according to the CIE L*a*b* system was performed by a blind trained operator at 5 experimental periods: immediately after light-polymerization (T0) and at 7 (T1), 14 (T2), 21 (T3), 28 (T4) days of the staining process. The control samples have not been subjected to the staining process. Before each measurement, the specimens were gently rinsed with distilled water and air-dried. Color of the specimens was measured with a spectrophotometer (SP820λ; Techkon Gmbh, Konig-Stein, Germany) against a black background in order to simulate the absence of light in the mouth against a white background. All specimens were chromatically measured 4 times and the average values were calculated; then each color parameter for each specimens of the same shade was averaged. The CIE 1976 L* a* b* color system is used for the determination of color differences (20,21). The L* value refers to “lightness”; the higher is the L value, and it is the lightness (a value of 100 corresponds to perfect white and that of zero to black). CIE L* a* b* values are called the “chromaticity coordinates”; “a*” shows red color on positive values and green color on negative values; “b*” shows yellow color on positive values and blue color on negative values (22) The total color differences (ΔEab*) were calculated as follows: (Fig. 1).

where L* is lightness, a* is green-red component (-a* = green; +a* = red) and b* is blue-yellow component (-b* = blue; +b* = yellow). A value of ΔEab* < 3.3 was considered clinically acceptable in the present study (1,23,24). The color measurements of the experimental groups were compared with those of the control group.

-Statistical analysis

Differences in color change by the immersion protocols were calculated and a statistical analysis was performed using statistical software (Stata 12; College Station, TX, USA). Descriptive statistics that included mean, standard deviation, median, and minimum and maximum values were calculated for each CIE L*a*b* parameter. Shapiro Wilk test was applied to assess the normality of the distribution of each CIE L*a*b* parameter. A 2-way non-parametric analysis of variance test (Kruskal-Wallis ANOVA) was applied to determine whether significant differences existed among the groups. Mann-Whitney test was used as post-hoc. A preset alpha level of .05 was used for all statistical analyses. Adjunctive analysis with paired t-t was applied to each CIE L*a*b* parameter when restorative materials were immersed in coffee.

## Results

**Figure 2:**
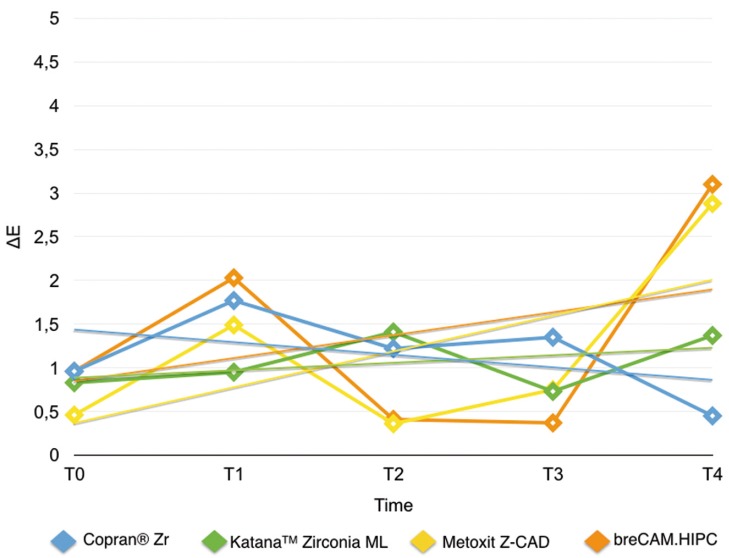
Evolution of the color variation ΔE for each material over the course of the study when immersed in physiological solution (negative control). Spectrophotometric analysis was made immediately after light-polymerization (T0) and at 7 (T1), 14 (T2), 21 (T3), 28 (T4) days.

**Figure 3:**
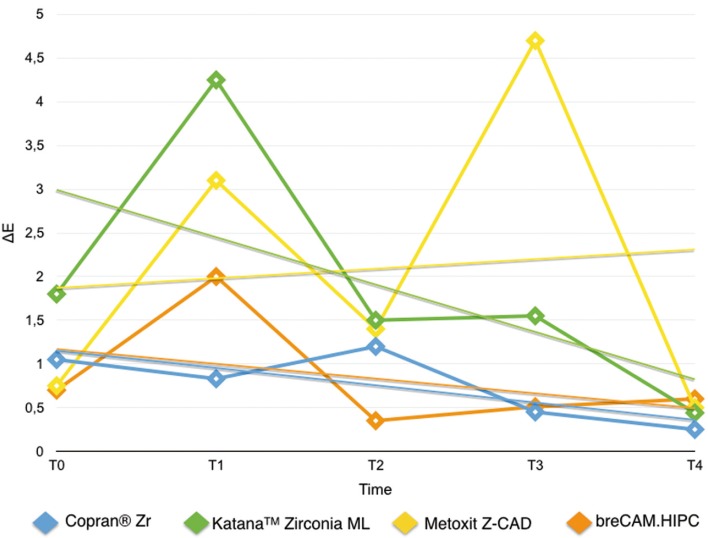
Evolution of the color variation ΔE for each material over the course of the study when immersed in acidic drink (Coca-Cola, The Coca-Cola Company, Milano, Italy) for 1 day + immersion in coffee. Spectrophotometric analysis was made immediately after light-polymerization (T0) and at 7 (T1), 14 (T2), 21 (T3), 28 (T4) days.

**Figure 4:**
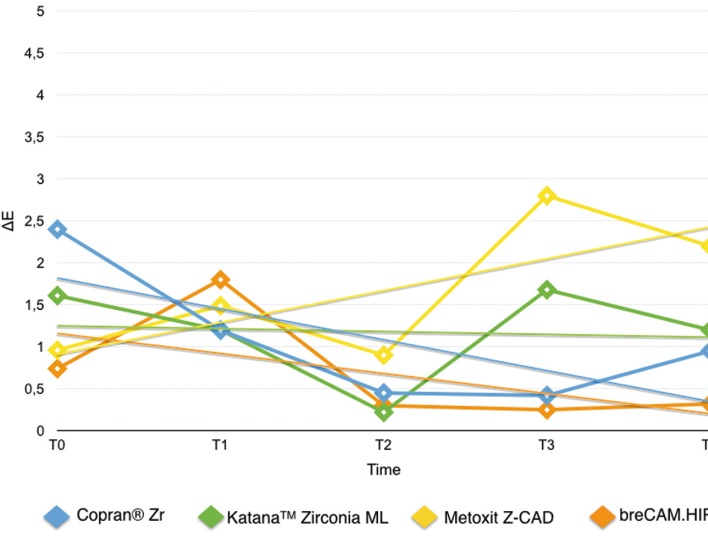
Evolution of the color variation ΔE for each material over the course of the study when immersed in acidic drink (Coca-Cola, The Coca-Cola Company, Milano, Italy) for 1 day + immersion in physiological solution. Spectrophotometric analysis was made immediately after light-polymerization (T0) and at 7 (T1), 14 (T2), 21 (T3), 28 (T4) days.

**Figure 5:**
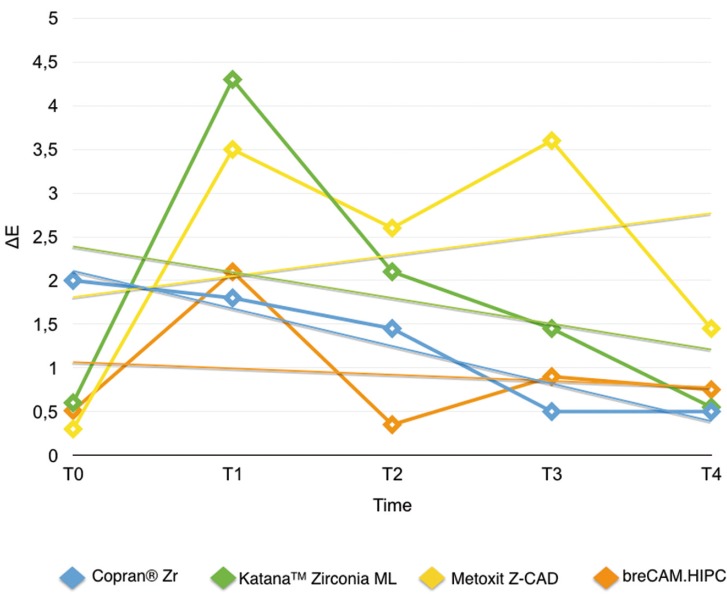
Evolution of the color variation ΔE for each material over the course of the study when immersed in acidic drink (Coca-Cola, The Coca-Cola Company, Milano, Italy) for 1 day + immersion in coffee. Spectrophotometric analysis was made immediately after light-polymerization (T0) and at 7 (T1), 14 (T2), 21 (T3), 28 (T4) days.

Results are summarized in  Table 2. Shapiro Wilk test confirmed that the values were not normally distributed. Kruskal Wallis ANOVA found significant differences among the various restorative materials. After one week immersion in control (T0-T1/Fig. 2) Copran® Zr, KatanaTM Zirconia ML and breCAM.HIPC showed similar mean discoloration, while lower clinically perceivable differences were recorded for Metoxit Z-CAD. Similar results were obtained with the immersion in physiologic solution except for Copran® Zr that showed higher color change after one week (*P* < 0.05). One week immersion in acidic drink (Fig. 3) did not cause a perceivable discoloration for all restorative materials (ΔE < 3.3). Subsequent immersion in coffee (Fig. 4) caused a clinically perceivable discoloration for KatanaTM Zirconia ML (ΔE > 3.3) after one week, while Metoxit Z-CAD showed a significant discoloration after three weeks of immersion in coffee. Immersion in coffee (Fig. 5) affected color stability of KatanaTM Zirconia ML and Metoxit Z-CAD also when specimens were firstly immersed in physiologic solution, as showed in Figs. 2-5. The restorative material breCAM.HIPC showed in all test groups a significant initial not perceivable discoloration after 1 week (T1), while the colorimetric evaluations at 2, 3 and 4 weeks showed a significantly lower ΔE.

**Table 2:**
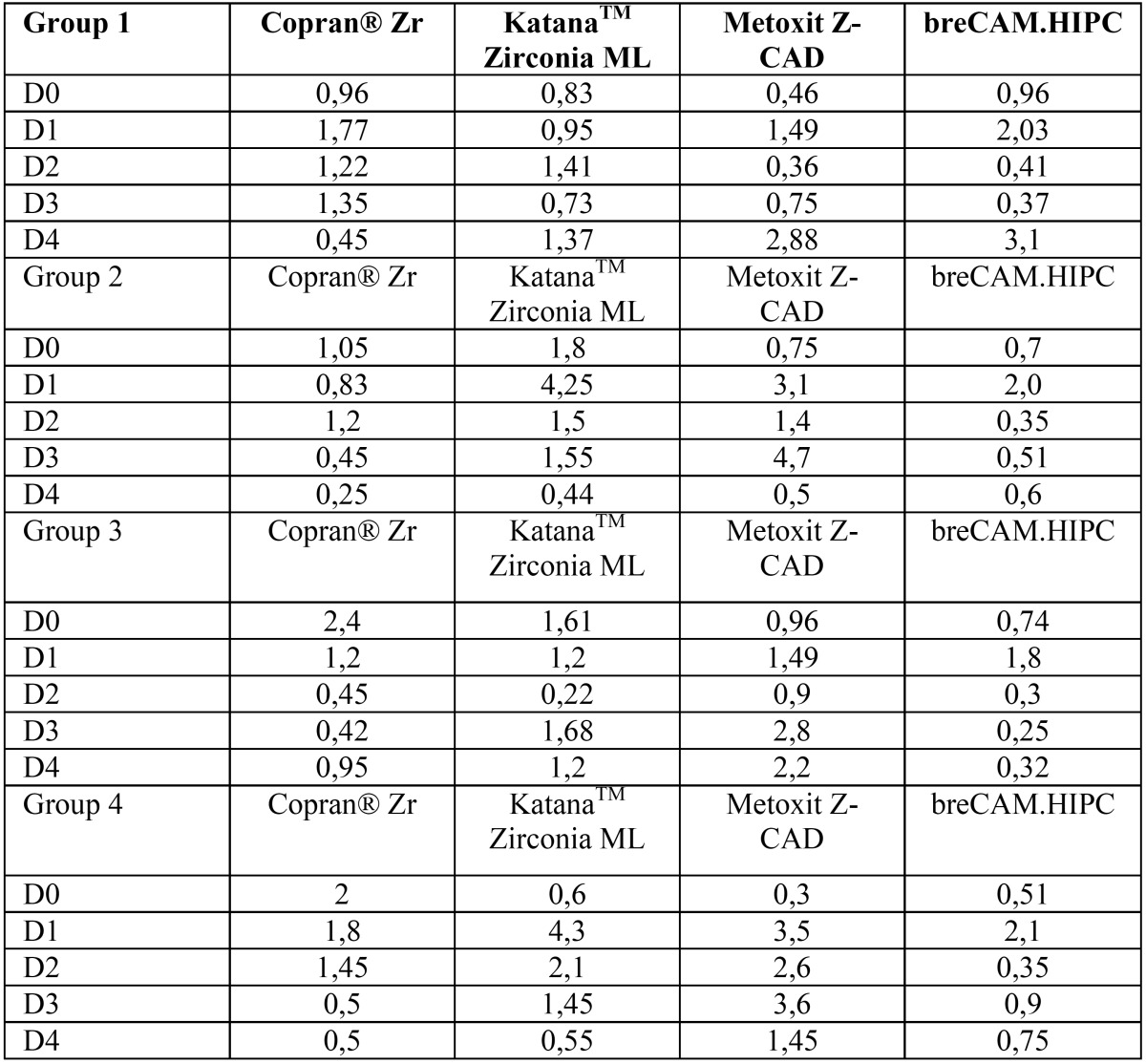
Mean ΔE for each CAD/CAM restorative material at each interval time. Same superscript letters reported in row mean no significant differences between CAD/CAM restorative materials (*P* > 0.05).

## Discussion

Restorations in the oral cavity are exposed to several factors that make them vulnerable to colour changes, such as temperature, humidity, food and smoking habits (17). In the oral environment, restorative materials are also subjected to numerous other liquids, to temperature and load stress, and to tooth brushing. The success of restorations depends not only on mechanical and physical properties, but also on the esthetic appearance (22).

Dental porcelain, combining wear resistance, strength, toughness and excellent esthetics, is considered to be the reference material for prosthetic rehabilitation, but composite resins have been widely used since their introduction because of their ease of handling in the evaluation and luting procedures (23). CAD/CAM processed composite resins were developed as alternatives to the ceramic blocks.

Based on the results of the present study, the immersion in coffe caused a general perceivable discoloration of the samples. The ∆E values after immersion in water were not significantly different, in contrast to those after immersion in coffee that showed a significant increase, This finding is in agreement with a previous study on luting agent which showed greater ∆E values of composite resin cements compared to PMMA-based resin cements (24).

According to Gawriołek *et al.* (25), ceramic materials exhibit better color stability than composite resins, and the results of the present study are in agreement.

Measuring color differences (ΔE) is commonly done in terms of the numerical distance in color space between the L*a*b* coordinates. If the ΔE value of two objects is less than 1 unit (ΔE < 1), then objects are judged to match. ΔE values from 1 to 3.3 units are important differences, but yet clinically considered acceptable (26). ΔE values more than 3.3 are perceptible by even untrained observers (e.g., patients), and not acceptable (27).

All tested materials showed ΔE values generally from 1 to 3.3 units, except for few cases, so the behavior of all Zirconia samples may be considered acceptable. Staining upon immersion in coffee was predominantly extrinsic, but the discoloration of CAD/CAM Zirconia blocks could be effectively removed with prophylaxis paste polishing, while that of some restorative composites could not be removed.

Previous literature suggested that immersion in coffee for one week was equivalent to seven months of coffee drinking with the assumption that the coffee remained in the mouth during drinking (24). Thus, one month immersion of specimens in coffee for evaluation of the resulting staining effect might be an exaggeration of the reality. It is assumed that regular oral hygiene measures can eliminate or reduce surface stains effectively.

A limitation of this study is that it was an in vitro study to allow for staining on both sides of the material. In a clinical situation, the material is bonded to a tooth structure and is exposed to solutions and light on only 1 side. Also, the color of the specimens is the combination of the gray background and the specimen’s color, and the color coordinate values may change in situations where different backgrounds are used. The results of this study should be corroborated with clinical studies. Further clinical and in vitro studies are necessary to evaluate the susceptibility of hybrid dental ceramic and resin nano-ceramic materials to discoloration by other beverages and nutrients.

## Conclusions

Within the limitations of the present study, the ∆Es of tested CAD/CAM Zirconia ceramics varied among the products after immersion in coffee, but their color integrity is not affected by contact/immersion in acidic drinks.
